# Association between Pan-Immune-Inflammation Value and Contrast-Induced Nephropathy with Coronary Angiography

**DOI:** 10.3390/medicina60061012

**Published:** 2024-06-20

**Authors:** Suleyman Akkaya, Umit Cakmak

**Affiliations:** 1 Department of Cardiology, Health Sciences University, Gazi Yasargil Research and Training Hospital, 21100 Diyarbakir, Turkey; 2 Department of Nephrology, Health Sciences University, Gazi Yasargil Research and Training Hospital, 21100 Diyarbakir, Turkey; umitccakmak@gmail.com

**Keywords:** coronary angiography, contrast-induced nephropathy, inflammation, pan-immune-inflammation value

## Abstract

*Background*: Contrast-induced nephropathy (CIN) is one of the most important complications after invasive cardiovascular procedures. Considering the pivotal role of inflammation in CIN development, the use of peripheral blood-based indexes may be an easily available biomarker to predict CIN risk. Therefore, in the present study, we evaluated the association between the pan-immune-inflammation value (PIV) and the risk of CIN. *Patients and Methods*: A total of 1343 patients undergoing coronary angiography (CAG) were included. The PIV was calculated with the following equation: (neutrophil count × platelet count × monocyte count)/lymphocyte count. Multivariable regression analyses were used to determine the association between clinical and laboratory parameters and CIN development. *Results*: The median age of the cohort was 58 (IQR 50–67), and 48.2% of the patients were female. CIN developed in 202 patients (15%) in follow-up. In multivariate analyses, older age (OR: 1.015, 95% CI: 1.002–1.028, *p* = 0.020) and higher PIV levels (OR: 1.016, 95% CI: 1.004–1.028, *p* = 0.008) were associated with a higher CIN risk, while the use of antiplatelet agents was associated with a lower risk of CIN (OR: 0.670, 95% CI: 0.475–0.945, *p* = 0.022). *Conclusions*: We demonstrated that the risk of CIN was significantly higher in patients with higher PIV and older patients in a large cohort of patients undergoing CAG for stable ischemic heart disease. If supported with prospective evidence, PIV levels could be used as a minimally invasive reflector of CIN.

## 1. Introduction

Contrast-induced nephropathy (CIN) is a unique kind of kidney damage resulting from exposure to iodinated contrast for diagnostic and therapeutic purposes [1]. While the incidence of CIN with diagnostic imaging in the general population was around one to two percent, CIN is still the third most common acute kidney injury (AKI) in hospitalized patients [2,3,4]. Previous research has shown that patients undergoing coronary angiography had over 3% CIN risk, possibly due to intra-arterial CIN use and the presence of comorbidities in these patients [5,6]. Additionally, patients undergoing percutaneous coronary intervention had CIN rates over 15% in several studies [7,8], pointing out a need for higher CIN awareness in patients with cardiovascular diseases undergoing CAG.

Historically, intravenous iodinated contrast media used for computed tomography imaging were considered major contributors to CIN [9]. However, contemporary comprehensive retrospective studies and meta-analyses have reassessed this paradigm, revealing no direct correlation between the administration of intravenous contrast media and acute kidney injury in a broad spectrum of patient populations [10,11]. In 2020, the American College of Radiology and the National Kidney Foundation released a consensus statement after reassessing the latest evidence on contrast-induced acute kidney injury (CI-AKI) and contrast-associated AKI (CA-AKI). This statement moderated the previously stringent guidelines regarding the administration of intravenous contrast media in patients with chronic, stable renal insufficiency [12]. It highlighted that a stable estimated glomerular filtration rate (eGFR) remains the most reliable indicator for assessing the risk of CI-AKI. The statement recommended increased vigilance in administering contrast media to patients not undergoing dialysis who have a stable eGFR below 30 mL/min/1.73 m² or those with fluctuating renal function, including those with existing AKI. However, it also acknowledged the limited evidence regarding the aggravation of pre-existing AKI via intravenous contrast media. Although this evidence-based guidance has been well-received, its application in cardiology is challenging due to the prevalence of patients with unstable renal function, including those with AKI [13].

There are several well-known risk factors for CIN. These include patient-related factors such as older age, presence of hypertension, diabetes, and chronic kidney disease and procedure-related factors like the use of intra-arterial contrast, high volume contrast use in therapeutic procedures, and the use of contrast with higher molecular weight [1,14,15]. While the main mechanism of CIN was suggested as the hemodynamic disturbance secondary to vasoconstriction, recent studies have pointed out the importance of inflammatory status secondary to increased oxidative stress and direct toxic effects of contrast media on tubular epithelium [16,17].

Considering the importance of inflammation on CIN development, several recent studies evaluated the association between the levels of immune-inflammatory parameters and CIN risk [18,19]. The use of peripheral blood-based indexes like neutrophil–lymphocyte ratio (NLR) and the systemic immune-inflammation index were reported to be easily available biomarkers to predict CIN risk [20]. However, no studies were completed to date with pan-immune-inflammation value (PIV), a novel compound inflammatory biomarker derived from the complete blood count. The PIV incorporates the levels of four immune-inflammatory cells and is suggested to be a better predictive biomarker than NLR due to its more comprehensive nature [21,22]. Therefore, in this study, we evaluated the association between PIV levels and the risk of CIN.

## 2. Materials and Methods

### 2.1. Study Population

This single-center, retrospective cohort study included patients aged over 18 years of age who underwent CAG for suspected stable ischemic heart disease between January 2020 and January 2021. All patients treated within prespecified dates, other than patients with chronic kidney disease, patients with decompensated heart failure, and patients who had missing creatinine levels at 48–72 h after CAG, were included in the study. Baseline clinical, laboratory, and angiographic data of all patients were retrospectively analyzed. The diagnosis of CIN was defined as either a 25% increase from baseline or a 0.5 mg/dL increase in serum creatinine levels observed 48–72 h after CAG [23]. In all procedures, nonionic and low osmolar contrast agents (iohexol, Omnipaque 350 mg/mL; GE Healthcare, Cork, Ireland) were utilized. Directly after CAG, an infusion of 0.9% NaCl solution at a rate of 1 mL/kg/h was administered for the first 12 h.

The following baseline clinical and laboratory parameters were retrieved from the patient files and electronic hospital registry: patient age, gender, baseline creatinine levels, creatinine levels at 48–72 h after CAG, neutrophil, platelet, monocyte, and lymphocyte counts from the baseline complete blood count, presence of diabetes and hypertension, and the use of calcium channel blockers, beta blockers, renin-angiotensin-aldosterone system (RAAS) blockers, statins, and antiplatelet agents. Additionally, the creatinine levels at three months after CAG were recorded. The data for the PIV score were collected from the complete blood count examinations retrieved in the preceding three days before the CAG procedure. The PIV was calculated with the following equation: [neutrophil count (10^3^/mL) × platelet count (10^3^/mL) × monocyte count (10^3^/mL)]/lymphocyte count (10^3^/mL).

### 2.2. Statistical Analyses

Descriptive statistics were presented as the median, interquartile range (IQR; 25th–75th percentile), and standard errors for continuous variables and frequency and percentages for categorical variables. The patients were categorized into CIN and no-CIN groups, and independent group comparisons were made using Mann–Whitney U and Chi-square tests for continuous and categorical variables, respectively. The association between clinical and laboratory parameters and CIN development was evaluated with multivariate analyses. Multivariate analyses were conducted using logistic regression analyses via backward variable selection, and odds ratios were calculated together with 95% confidence intervals (CI). Clinical and laboratory parameters with *p*-values < 0.10 were included in the multivariate model. Due to the adequate sample size, the laboratory parameters were used as continuous parameters in multivariate analyses without a cut-off selection to better delineate the precise risk increase for CIN. All statistical analyses were performed in SPSS, version 25.0 (IBM Inc., Armonk, NY, USA), and a *p* value of less than 0.05 was considered statistically significant.

## 3. Results

A total of 1343 patients were included in the analyses; 48.2% of the patients were female, and the median age of the cohort was 58 (IQR 50–67). Diabetes, hypertension, and heart failure were present in 20.7%, 35.6%, and 14.2% of the patients, respectively. A total of 31.9% of the patients were using renin-angiotensin-aldosterone system (RAAS) inhibitors, while beta-blocker use was present in 30.9% of the patients (Table 1).

CIN developed in 202 patients (15%) in follow-up. The presence of hypertension, diabetes, and heart failure did not have a significant association with the development of CIN (*p* > 0.05 for each). Similarly, the use of RAAS inhibitors, statins, and beta-blockers did not have a significant association with CIN, while there was a trend towards lower CIN risk in patients regularly taking antiplatelet agents (16.2% vs. 12.7%, *p* = 0.093). The median age and median albumin and CRP levels were similar across the two groups, while patients who developed CIN had higher median NLR and PIV levels (Table 2). There was also no significant difference between the two groups regarding the baseline creatinine and Glomerular Filtration Rate (GFR).

A multivariate model was created using antiplatelet agents, NLR, and PIV levels. Age was also added to the multivariate model as a continuous parameter. PIV levels were divided by 100 before using them in the multivariate model. In multivariate analyses, older age (OR: 1.015, 95% CI: 1.002–1.028, *p* = 0.020) and higher PIV levels (OR: 1.016, 95% CI: 1.004–1.028, *p* = 0.008) were associated with a higher CIN risk, while the use of antiplatelet agents was associated with a lower risk of CIN (OR: 0.670, 95% CI: 0.475–0.945, *p* = 0.022). The association between NLR levels and CIN risk did not reach statistical significance (Table 3).

## 4. Discussion

In the present study, we observed significantly higher CIN risk in patients with higher PIVs and older patients, while the patients who were using antiplatelet agents had a lower risk of CIN. Every 100 unit increase in PIV levels was associated with a 1.6% risk increase for CIN. To our knowledge, our study is the first study in the literature reporting an association between PIV levels and CIN risk.

The use of peripheral blood count-based compound immune-inflammatory indexes in the risk and prognosis prediction of diseases with an inflammation background has gained a lot of interest in the last decade [24,25]. These indexes are mainly based on dividing the number of cells that are related to inflammatory pressure, like neutrophils and platelets, by the number of lymphocytes, the main driver of adaptive immunity and the brake for uncontrolled inflammation [26,27]. The NLR is the most frequently tested index in this regard; although, it has often been criticized for including only two parameters from the peripheral blood cells [28]. The PIV score was developed and validated three years ago in patients with colorectal cancer with the aim of including more immune-inflammatory peripheral blood cells to improve precision [23]. In fact, several studies have demonstrated that PIV could be a better biomarker than NLR, including the pivotal PIV study by Fuca et al. [29]. Similarly, in our study, we observed that in a multivariate model, PIV had a statistically significant association with CIN risk, while the association between NLR and CIN risk did not reach statistical significance, supporting a broader use of PIV as an immune-inflammatory index instead of NLR.

There are many risk warning systems for CIN, and different models have a variety of predictors and risk thresholds for the same predictor. The Mehran score, established by Mehran et al. in 2004, is a classic and the most commonly used scoring system; but it still has some flaws [30]. For example, it contains up to eight variables, including perioperative variables, which greatly reduce the system’s clinical application ability, especially for patients who need PCI surgery due to acute myocardial infarction, with a C-statistic of only 0.67; thus, standard risk scores developed in patient cohorts nearly 20 years ago may no longer be the most accurate option. The model was updated in 2021 by Mehran et al. [31]. The updated model is an AKI risk-prediction score based on data from 14,616 PCI patients in the United States, including Model 1 with preoperative variables and Model 2 with additional postoperative variables. Compared with the original Mehran score, there was higher discrimination, and patients with STEMI were included. The Mehran 2 score exhibited enhanced discriminatory capabilities compared to its predecessor and incorporated considerations for patients presenting with ST-elevation myocardial infarction (STEMI). This refinement allows for more accurate risk predictions in contemporary percutaneous coronary intervention (PCI) cohorts, potentially facilitating a reduction in the incidence of adverse outcomes. It is important to note that, in our study, the Mehran score was not calculated prior to the procedure. This reflects a gap in the comprehensive risk assessment that might have provided additional insights into the CIN risk stratification.

Older age is an important risk factor for CIN, as seen in our study [32]. While the risk of CIN was reported to be over 5% percent in large studies, patients over 75 years of age undergoing CAG were among the highest risk for CIN [18,33]. Furthermore, older age was among the clinical parameters that was used in the eigth-item CIN score [1]. The age-related changes in the kidneys, as well as the additional comorbidities, were suggested as the reasons for higher CIN risk in older patients. An interesting finding in our study was the lower CIN risk in patients with regular antiplatelet use. While aspirin was regarded as safe for renal function, even when regularly used for long periods, a CIN-protective role of antiplatelet agents was not demonstrated before, necessitating evaluation in prospective cohorts before reaching a conclusion. The possibility of better microvasculature and lower inflammatory pressure could be reasons for this result, although the median PIV and NLR levels were similar between patients who were taking antiplatelet agents and those who were not.

Our study has several limitations inherent to retrospective study design and the study sample. Although all patients underwent CAG in accordance with the recent international guidelines, the differences stemming from the contrast volume could not be excluded. Additionally, the study cohort exhibited a relatively younger age, limiting the generalizability of the results. However, despite these limitations, we think that our findings point out the possibility of using PIV as a risk factor for CIN, if supported with further prospective evidence.

## 5. Conclusions

In conclusion, we observed significantly higher CIN risk in patients with higher PIV scores and older patients in a large cohort of patients undergoing CAG for stable ischemic heart disease. Further research is needed to validate the use of PIV as a risk factor for CIN and to aid in the treatment and follow-up decisions for CIN.

## Figures and Tables

**Table 1:** Demographic, clinical, and laboratory features of the participants.

Baseline Characteristics	
Age (years), median (IQR)	58 (50–67)
Female gender, n (%)	696 (48.2)
Diabetes mellitus, n (%)	278 (20.7)
Hypertension, n (%)	478 (35.6)
Heart failure, n (%)	191 (14.2)
Medications prior to CAG	
Antiplatelet agents, n (%)	441 (32.8)
ACEI/ARB, n (%)	429 (31.9)
Beta blockers, n (%)	415 (30.9)
Calcium channel blocker, n (%)	165 (12.3)
Statin, n (%)	170 (12.7)
Nitrates, n (%)	0 (0)

**Table 2:** Comparison of the baseline characteristics and laboratory findings of the two groups.

	With CIN (n = 202)	Without CIN (n = 1141)	*p* Value
Age (years), median (IQR)	60 (51–69.25)	58 (50–67)	0.787
Female gender, n (%)	103 (51.0)	544 (47.7)	0.385
Diabetes mellitus, n (%)	47 (23.3)	231 (20.2)	0.329
Hypertension, n (%)	76 (37.6)	402 (35.2)	0.513
Heart failure, n (%)	30 (14.9)	161 (14.1)	0.781
Medications prior to CAG			
Antiplatelet agents, n (%)	56 (27.7)	385 (33.7)	0.093
ACEI/ARB, n (%)	66 (32.7)	363 (31.8)	0.809
Beta blockers, n (%)	67 (33.2)	348 (30.5)	0.449
Calcium channel blocker, n (%)	20 (9.9)	145 (12.7)	0.263
Statin, n (%)	24 (11.9)	146 (12.8)	0.719
Laboratory findings			
Serum creatinine (mg/dL), median (IQR)	0.76 (0.63–0.88)	0.77 (0.67–0.92)	0.301
eGFR (mL/min/1.73 m^2^)	87 (77–99)	90 (77–102.25)	0.132
Haemoglobin (g/dL), median (IQR)	13.77 (12.17–15.20)	13.90 (12.50–15.10)	0.542
White cell count (10^3^/mL), median (IQR)	8.71 (7.21–10.36)	8.47 (7.09–10.39)	0.772
Neutrophil count (10^3^/mL), median (IQR)	6.06 (4.76–8.03)	5.24 (4.09–6.78)	<0.001
Lymphocyte count (10^3^/mL), median (IQR)	2.00 (1.38–2.85)	2.16 (1.56–2.79)	0.490
Monocyte count (10^3^/mL), median (IQR)	0.56 (0.44–0.71)	0.52 (0.41–0.66)	0.015
Platelet count (10^3^/mL), median (IQR)	270 (213–323)	255 (213–299)	0.062
HDL-C (mg/dL), median (IQR)	41.50 (35.07–49.55)	43 (36.30–50.50)	0.258
LDL-C (mg/dL), median (IQR)	106.61 (80.00–131.50)	108.00 (86–133)	0.358
Triglycerides (mg/dL), median (IQR)	160 (103–243.50)	141 (99–212)	0.052
Albumin (g/dL), median (IQR)	4 (3.37–4.30)	4 (3.50–4.30)	0.454
CRP (mg/L), median (IQR)	2.70 (2.00–10.02)	2.00 (2.00–6.20)	0.130
NLR, median (IQR)	2.96 (1.85–4.55)	2.47 (1.70–3.73)	0.001
PIV, median (IQR)	444.86 (262.46–708.61)	329.91 (195.08–553.31)	<0.001

Abbreviations: HDL: high-density lipoprotein; IQR: interquartile range; LDL: low-density lipoprotein; NLR: neutrophil-to-lymphocyte ratio; PIV: pan-immune-inflammation value.

**Table 3:** Univariate and multivariate logistic regression analysis for presence of CIN.

	Univariate Analysis			Multivariate Analysis		
Variable	OR	95% CI	*p*-Value	OR	95% CI	*p*-Value
Age (continuous)	1.013	1.000–1.025	0.050	1.015	1.002–1.028	0.020
Antiplatelet agents	0.753	0.541–1.049	0.094	0.670	0.475–0.945	0.022
NLR	1.024	1.005–1.045	0.016	1.010	0.985–1.036	0.415
PIV/100	1.016	1.004–1.028	0.008	1.016	1.004–1.028	0.008

## Data Availability

The datasets used and/or analyzed during the current study are available from the corresponding author on reasonable request.
